# The experiences of caregivers of children with epilepsy: A meta-synthesis of qualitative research studies

**DOI:** 10.3389/fpsyt.2022.987892

**Published:** 2022-09-13

**Authors:** Zhichao Yu, Qinwen Shao, Kunhua Hou, Yanjie Wang, Xianghong Sun

**Affiliations:** ^1^Shengjing Hospital of China Medical University, Shenyang, China; ^2^Wuhan University of Bioengineering, Wuhan, China; ^3^Henan Provincial People's Hospital, Zhengzhou, China; ^4^China Medical University, Shenyang, China

**Keywords:** experience, caregiver, children, epilepsy, burden of care, qualitative study, meta-synthesis

## Abstract

**Objective:**

Epilepsy is one of the most common chronic neurological disorders in children. The caregivers of these children bear heavy burden of care in the process of taking care of them. The objective of this metasynthesis was to explore the experiences and needs of caregivers of children with epilepsy.

**Methods and data sources:**

Eight databases (PubMed, CINAHL, EMBASE, Web of Science, CNKI, Wanfang Data, VIP database, and CBM) were searched for qualitative studies from each database's inception to 31 June 2021. Studies were critically appraised using the Joanna Briggs Institute (JBI) Critical Appraisal Checklist for Qualitative Research. Qualitative data were extracted, summarized, and meta-synthesized.

**Results:**

13 studies were included, covering the data of 316 participants. 36 research results were extracted from these studies, which were combined into 11 categories, and finally formed 4 themes: (a) heavy burden of care; (b) emotional experience; (c) coping strategies; (d) care needs.

**Conclusion:**

Caregivers beared a heavy burden of care and psychological burden. Despite the adoption of different coping strategies, their emotional distress was still very serious. Caregivers had unmet care needs. In order to improve caregivers' care capacity, the society and healthcare workers need to provide them with information support, psychological support, and take measures to create a friendly medical and living environment for them.

**Impact:**

Understanding the experiences of caregivers of children with epilepsy will inform future research and practice. Healthcare workers could develop interventions to reduce caregiver burden and improve the level of caregivers' mental health. On the other hand, effective programs should be designed to improve caregivers' knowledge of the disease and enhance their ability to care. Society needs to take steps to improve the medical environment and the social stigma that is not friendly to epilepsy.

## Introduction

Epilepsy is one of the most common chronic neurological disorders in children (1), with an estimated global incidence of childhood epilepsy ranging from 41 to 187 per 100,000 people (2). Epilepsy is characterized by sudden onset, recurrence, long course of disease, long time of taking medication, and great drug side effects (3). Due to social stigma and prejudice, children with epilepsy have a higher incidence of psychological disorders. Studies have shown that nearly half of teenagers with epilepsy suffer from depressive symptoms. At the same time, 30~40% of epileptic patients have cognitive impairments, including daily learning, memory, attention and executive control (4), which seriously affect children's normal learning and social activities, reduce the quality of life of children and their families.

Due to the lack of self-management ability, caregivers are often faced with great challenges in the process of treatment and rehabilitation of children. Caregivers often experience uncertainty due to fear of seizures at any time and need to continuously monitor the child's status. In addition, parents need to cope with specific diets, activity restrictions, long-term medication and side effects, school and social challenges, and stigma (5). The psychological, behavioral, social, educational and cultural factors involved have a significant impact on the lives of children and their families (6). Previous studies have shown that nearly half of caregivers of children with epilepsy develop psychopathological symptoms, including post-traumatic stress disorder, depression, anxiety and high levels of stress, and they even had trouble sleeping (7). Caregivers' mental state and disease management ability are important factors affecting children's rehabilitation (8).

The experiences of caregivers of children with epilepsy are complex and heavy. Studies have shown that caregivers of children with epilepsy bear a heavy burden of care and psychological burden (9, 10). At the same time, caregivers' lack of disease management knowledge (11) indicates that caregivers' need for disease management knowledge is not being met. Therefore, it is necessary to understand the experiences and needs of caregivers of children with epilepsy in the process of care.

In recent years, a growing number of studies explored the experiences of caregivers of children with epilepsy (3, 8, 12–14), but there was no related systematic review or metathesis. Therefore, it is necessary to integrate the experiences and challenges of caregivers, and identify their care needs. This will enable policy makers to take targeted measures to improve caregivers' mental health, remove barriers to care, enhance care capacity, and ultimately promote children's recovery and development.

## The review

### Aims

To synthesize qualitative studies on the experiences of caregivers of children with epilepsy.

### Design

A systematic qualitative review and meta-synthesis was performed in this study. The process of meta-synthesis included analyzing, classifying, evaluating and summarizing the results of qualitative research. The protocol for this review has been registered (PROSPERO: CRD42021262770).

### Search methods

A systematic search strategy was carried out in June 2021. Eight English and Chinese databases were searched, including PubMed, CINAHL, EMBASE, Web of Science, CNKI, Wanfang Data, VIP database, and CBM. English search keywords included: “epilepsy” or “falling sickness” or “epilepsia” or “seizure” or “epileptic”; “caregivers” or “parent” or “take care” or “look after” or “nursing”; “qualitative study” or “qualitative research”; “experience” or “reaction” or “perception” or “need” or “feeling.” The language was limited to Chinese and English, and no date restrictions were applied to database searches. Further, references of discovered papers were also checked, and those that met the inclusion criteria were included in this study. The inclusion criteria were set based on PICo-D (participant, interest in phenomena, context and design). The detailed contents of PICo-D can be seen in Table 1.

**Table 1:** Review inclusion and exclusion criteria.

**Inclusion criteria**	**Exclusion criteria**
- P (population): Inclusion criteria: (1) Relatives of children with epilepsy who took care of the children every day to at least 4 hours;(2) To be able to express their care experience in words;(3) Volunteer to participate in this study.	- Exclusion criteria: paid daily care for the child - Papers not written in Chinese or English. - Papers with abstracts and without full texts.
- I (interest of phenomena): Experiences, feelings and needs in the process of caring for children with epilepsy.	- Duplicate records. - Papers with incomplete data.
- Co (Context): The experience of the caregiver of a child with epilepsy in taking care of the child in daily life.	
- D (design): Qualitative research, including phenomenology, grounded theory, ethnography and other qualitative research methods articles.	

### Search outcomes

**Figure 1:**
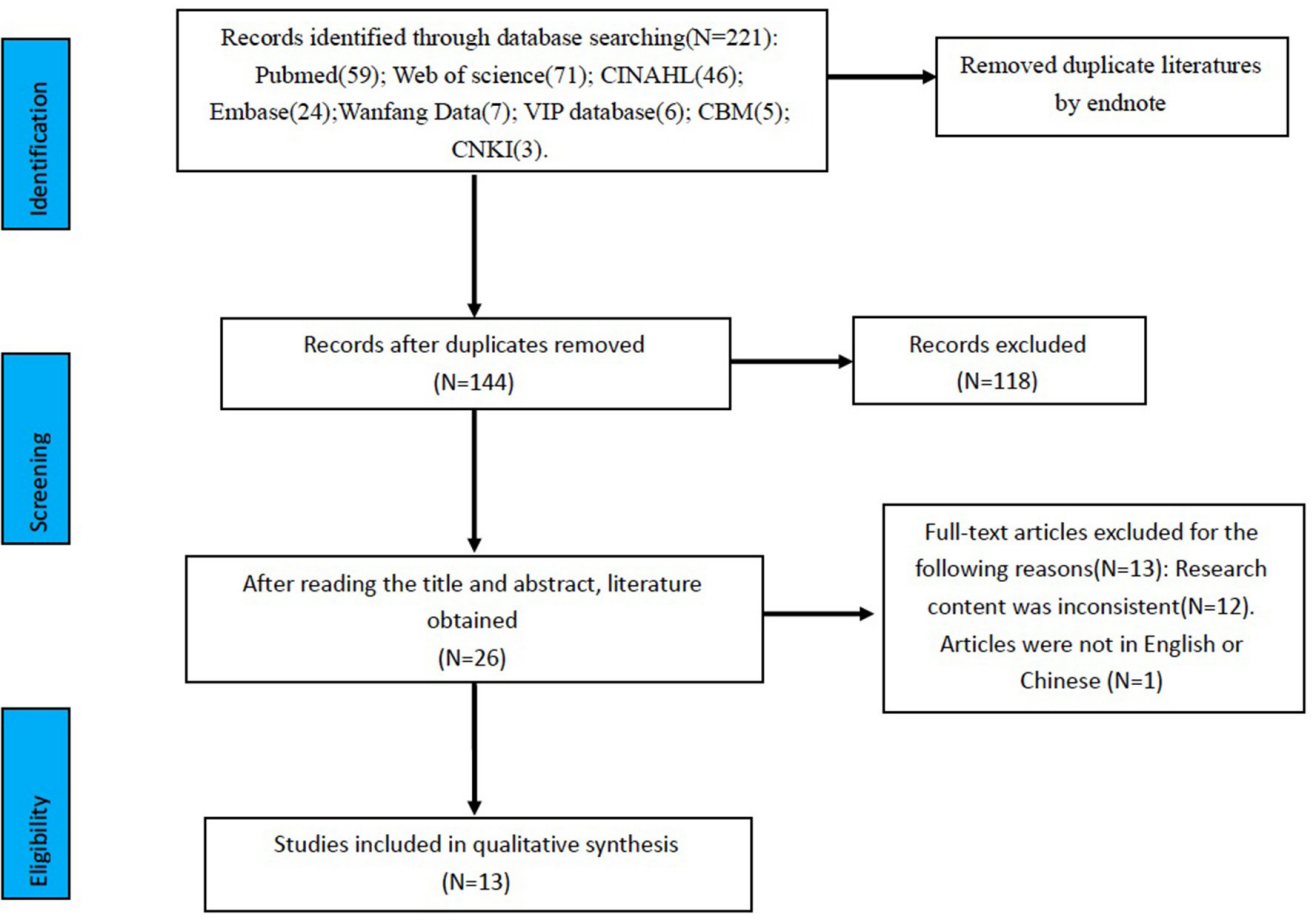
Flow diagram of literature selection.

The literatures retrieved in this review were imported in Endnote X9 programme (Clarivate Analytics) and duplicates were removed through it. Two reviewers independently read and evaluated the title, abstract and full text of the literatures according to the inclusion and exclusion criteria. If they were controversial during the literature screening process, a third reviewer would make the decision. A total of 221 literatures were retrieved from eight databases and 77 duplicates were excluded. After reading the title and abstracts according to inclusion and exclusion criteria, 118 unrelated articles were excluded. Then, 26 literatures were left and their full texts were read, and finally 13 literatures that met the research criteria were obtained. Figure 1 is the flow diagram of this review.

### Quality appraisal

Two reviewers independently assessed the qualitative literatures included in this review according to the Joanna Briggs Institute (JBI) Critical Appraisal Checklist. The items of the checklist included research methods and their philosophical perspectives, research objectives, methods of data collection, methods of data analysis, interpretation of results, statements of positioning the researcher from a cultural or theoretical perspective, and the researcher's influence on the research. Each evaluation item in the checklist included four options: yes, no, unclear, and not applicable. The quality of the included literatures was classified into three grades: A, B, and C. In case of disagreement among reviewers, discussion was held together, and if necessary, a third reviewer would decide whether to include the literature. Detailed quality assessment results are shown in Table 2.

**Table 2:** Methodological quality appraisal of the included studies.

**References**	**Item 1**	**Item 2**	**Item 3**	**Item 4**	**Item 5**	**Item 6**	**Item 7**	**Item 8**	**Item 9**	**Item 10**	**Overall appraisal**
**Wu et al**. **(****12****)**	Y	Y	Y	Y	Y	N	N	Y	U	Y	B
**Zhang et al**. **(****3****)**	Y	Y	Y	Y	Y	N	N	Y	Y	Y	B
**Smith et al**. **(****13****)**	Y	Y	Y	Y	Y	N	N	Y	Y	Y	B
**Wang et al**. **(****8****)**	Y	Y	Y	Y	Y	N	N	Y	Y	Y	B
**Wo et al**. **(****15****)**	Y	Y	Y	Y	Y	N	N	Y	Y	Y	B
**Amjad et al**. **(****16****)**	Y	Y	Y	Y	Y	N	N	Y	Y	Y	B
**Nguyen et al**. **(****17****)**	Y	Y	Y	Y	Y	N	N	Y	U	Y	B
**Murugupillai et al**. **(****18****)**	Y	Y	Y	Y	Y	N	N	Y	Y	Y	B
**O'Toole et al**. **(****19****)**	Y	Y	Y	Y	Y	N	N	Y	Y	Y	B
**Kampra et al**. **(****20****)**	Y	Y	Y	Y	Y	N	Y	Y	Y	Y	B
**Benson et al**. **(****21****)**	Y	Y	Y	Y	Y	N	N	Y	Y	Y	B
**Jones et al**. **(****14****)**	Y	Y	Y	Y	Y	N	Y	Y	Y	Y	B
**Amjad et al**. **(****22****)**	Y	Y	Y	Y	Y	N	N	Y	U	Y	B

Appraisal checlist: ① Is there congruity between the stated philosophical perspective and the research methodology? ② Is there congruity between the research methodology and the research question or objectives? ③ Is there congruity between the research methodology and the methods used to collect data? ④ Is there congruity between the research methodology and the representation and analysis of data? ⑤ Is there congruity between the research methodology and the interpretation of results? ⑥ Is there a statement locating the researcher culturally or theoretically? ⑦ Is the influence of the researcher on the research, and vice- versa, addressed? ⑧ Are participants, and their voices, adequately represented? ⑨ Is the research ethical according to current criteria or, for recent studies, and is there evidence of ethical approval by an appropriate body? ⑩ Do the conclusions drawn in the research report flow from the analysis, or interpretation, of the data?

Appraisal result: Y, Yes; N, No; U, Unclear; N/A, Not applicable.

### Data abstraction

Each study was thoroughly read by reviewers, and then useful data were extracted. The extracted content included author (year), country, aim, sample size, study design, data collection and data analysis, setting, and results. The details of the extraction are shown in Table 3.

**Table 3:** Description of included studies.

**References**	**Country**	**Journal**	**Aim**	**Sample size**	**Study design**	**Data collection and data analysis**	**Setting**	**Results**
Wu et al. (12)	China	Chinese Journal of Nursing	To explore the lived experience of primary caregivers of children with epilepsy	Twelve caregivers	Phenomenological research method	Data collection: Interviews Data analysis: LoBiondo-Wood Phenomenological analysis method	Ward	Four t hemes: Bearing the burden of care (the original rhythm of life has been disrupted, the health status of caregivers themselves is affected, economic overburden); Psychological overload (remorse and guilt, fear, anxiety and helplessness, uncertainty about the future); Lack of knowledge of family care (lack of knowledge of first aid, lack of knowledge about the disease, lack of ability to make treatment decisions); Weak support system (lack of family support systems, limited medical conditions, lack of social support systems)
Zhang et al. (3)	China	Qilu Nursing Journal	To understand the needs of nursing services in the process of out-of-hosptial home care for the main caregivers of children with epilepsy	Eleven caregivers	Phenomenological research method	Data collection: Interviews Data analysis: Colaizzi Phenomenological analysis method	Ward	Four themes: Information needs (epilepsy knowledge information needs, drug management information needs, the need for coping methods during an acute attack, the need for healthy living guidance); Psychological needs (psychological support need, family support needs); Need for social support
Smith et al. (13)	America	Epilepsy & Behavior	To explore caregivers' perceptions of the caregiving process at different time periods postepilepsy diagnosis	Nineteen caregivers	Qualitative research method	Data collection: Focus group Data analysis: Thematic analysis method	Unclear	Four themes: Navigating the non-contingencies, Blessings and sacrifices, Constant vigilance, Caregiving is more than parenting
Wang et al. (8)	China	Modern Nurse	To explore the home care needs of primary caregivers of children with epilepsy	Twelve caregivers	Descriptive qualitative research method	Data collection: Interviews Data analysis: Content analysis method	Reference room	Three themes: The need to acquire knowledge of the disease (the need for first aid knowledge, the need for medication knowledge, diet and activity guidance needs, the need for professional guidance, the need for disease treatment decisions); The need for psychological counseling (fear and anxiety, worry and remorse); The need to reduce the burden of care (the need to lighten your body's load, the need to lighten the financial burden, the need to relieve pressure on schools, the need to reduce the stress of medical care)
Wo et al. (15)	Malaysia	Epilepsy & Behavior	To explore the experiences of parents and their children, and to identify the needs and challenges faced by parents and children in childhood epilepsy care	Fifteen families	Descriptive phenomenology approach	Data collection: Interviews Data analysis: Thematic analysis method	Paticipants' home	Experiences during child's first seizure: Parents' initial reactions (emotional reactions to child's first seizure, causes of epilepsy, sociocultural role in health-seeking behavior) Experiences while growing up with epilepsy: Impact of epilepsy on the family (the positive impact on the family, the negative impact on the family); Management of epilepsy care (vigilance in caring for a child with epilepsy, parents' coping strategies, disclosure of epilepsy); Unmet parental needs (need for epilepsy-related information, need for continuity of care, need for a parental support group); Parents' perceived impact of epilepsy on their child (physical changes, emotional changes, behavioral changes, academic achievement, interpersonal relationship)
Amjad et al. (16)	Iran	Journal of Caring Sciences	To understand the experiences of parent of child with epilepsy in Iran	Ten parents	Interpretative phenomenological approach	Data collection: Interviews Data analysis: Van Manen's method	In a quiet room	Main theme: Family stigma Three subthemes: Becoming verbally abusive; A dull and heavy shadowed look; Associates interference
Nguyen et al. (17)	Australia	Clinical Child Psychologyand Psychiatry	To understand parents' internal narratives and experience of chronic illness in their child	Twenty mothers	Phenomenological research methods	Data collection: Interviews Data analysis: Theory-driven thematic analysis	Unclear	Three themes: adjustment process, (Experience promotes adaptation) cognitive appraisals (Normalizing epilepsy, Maintaining a positive focus, One day at a time, control, Meaning in adversity) and coping behaviors (Emotional ventilation, Problem-solving, Time to self, Speaking with other parents)
Murugupillai et al. (18)	Sri Lanka	Seizure	To identify the parental concerns regarding their children and adolescents with epilepsy in Sri Lanka	The parents of sixteen children with epilepsy and Four primary health care members	Qualitative study	Data collection: Interviews Data analysis: Content analysis	Home and workplace	Concern about physical functioning, Concern about behavioral and cognitive functioning, Concern about education, Concern about psychological/emotional functioning, Concern about social functioning, Concern about epilepsy in general and Concern about treatment with anti-epileptic medicines
O'Toole et al. (19)	Ireland	Epilepsy & Behavior	To explore the challenges that parents of children with epilepsy experienced when engaging in dialog with their child about epilepsy and epilepsy-related issues	Thirty-four parents	Qualitative study	Data collection: Interviews Data analysis: Braun and Clarke's six-step thematic analysis	The place Convenient for participants	Normalizing epilepsy, the invisibility of epilepsy, information concealment, fear of misinforming the child, and difficulty in discussing particular epilepsy-related issues
Kampra et al. (20)	Greece	Epilepsy & Behavior	To explore the challenges that Greek parents/caregivers of children with controlled epilepsy (CwE) face regarding the disorder	Ninety one parents/caregivers	Phenomenological research methods	Data collection: Interviews Data analysis: Van Manen's process	Hospital	The disclosure of epilepsy (How can I explain epilepsy to my child, Why should I inform the school staff about my child's epilepsy, Why should I tell anyone about my child's epileps), Absence of adequate information about coping with epilepsy (Where could we seek help to cope with our child's epilepsy after our visit to the doctor, No expert support in regular schools)
Benson et al. (21)	Ireland	Patient Education and Counseling	Aim to present the stigma experiences of children with controlled epilepsy and their parents, in the context of communicating about epilepsy within and external to the family unit	Thirty children with controlled epilepsy and fourty parents of children with controlled epilepsy	Mixed-methods sequential exploratory design	Data collection: Interviews Data analysis: Braun and Clarke's six-step thematic analysis	Unclear	Concealment (the potential for stigmatization due to epilepsy), stigma-coaching (parents' perceiving seizures Negatively)
Jones et al. (14)	America	Epilepsy & Behavior	To understand parents' needs, values, and preferences to ultimately reduce barriers that may be impeding parents from accessing and obtaining help for the child's co-occurring problems	Twenty-two parents	A qualitative study	Data collection: Interviews Data analysis: Grounded theory approach	Unclear	Describe their concerns about the child's struggles, their understanding of the struggles, and the parent's view of the child's future
Amjad et al. (22)	Iran	Acta Medical Mediterranea	Aimed at exploring experience of parents of children with epilepsy	Fourteen parents	Phenomenological research methods	Data collection: Interviews Data analysis: Van Manen's process	A quiet room	Fenced in by the child disease (Limitation in relationships, travel restrictions, drop out from school, leaving the job)

### Synthesis

In this review, a meta-synthesis method (23) was adopted to integrate the results of qualitative studies. Each study was read and re-read to ensure familiarity with the content. Two reviewers, respectively collected the research results including the theme, implied meaning, category, etc., and then integrated and summarized the research results according to their meanings to make them targeted, persuasive and general. On the premise of understanding the philosophical thought and methodology of qualitative research, reviewers repeatedly read, analyzed and interpreted the previous research results, summarized and combined similar results to form new genera, and then summarized the new genera into integrated results. Any discrepancies that arose were discussed between the two reviewers until a consensus was reached.

## Results

### Study characteristics

This qualitative meta- synthesis included a total of 13 studies, with 316 participants from eight different countries and cultures: China (*N* = 3) (3, 8, 12), America (*N* = 2) (13, 14), Malaysia (*N* = 1) (15), Ireland (*N* = 2) (19, 21), Iran (*N* = 2) (16, 22), Australia (*N* = 1) (17), Sri Lanka (*N* = 1) (18), Greece (*N* = 1) (20) (Table 3). Among the 13 included studies, 12 used phenomenological research methods and one study Jones et al. (14) used grounded theory methods. Whilst one study (18) used focus group discussions, the other 12 studies used interviews for data collection.

### Qualitative synthesis

**Figure 2:**
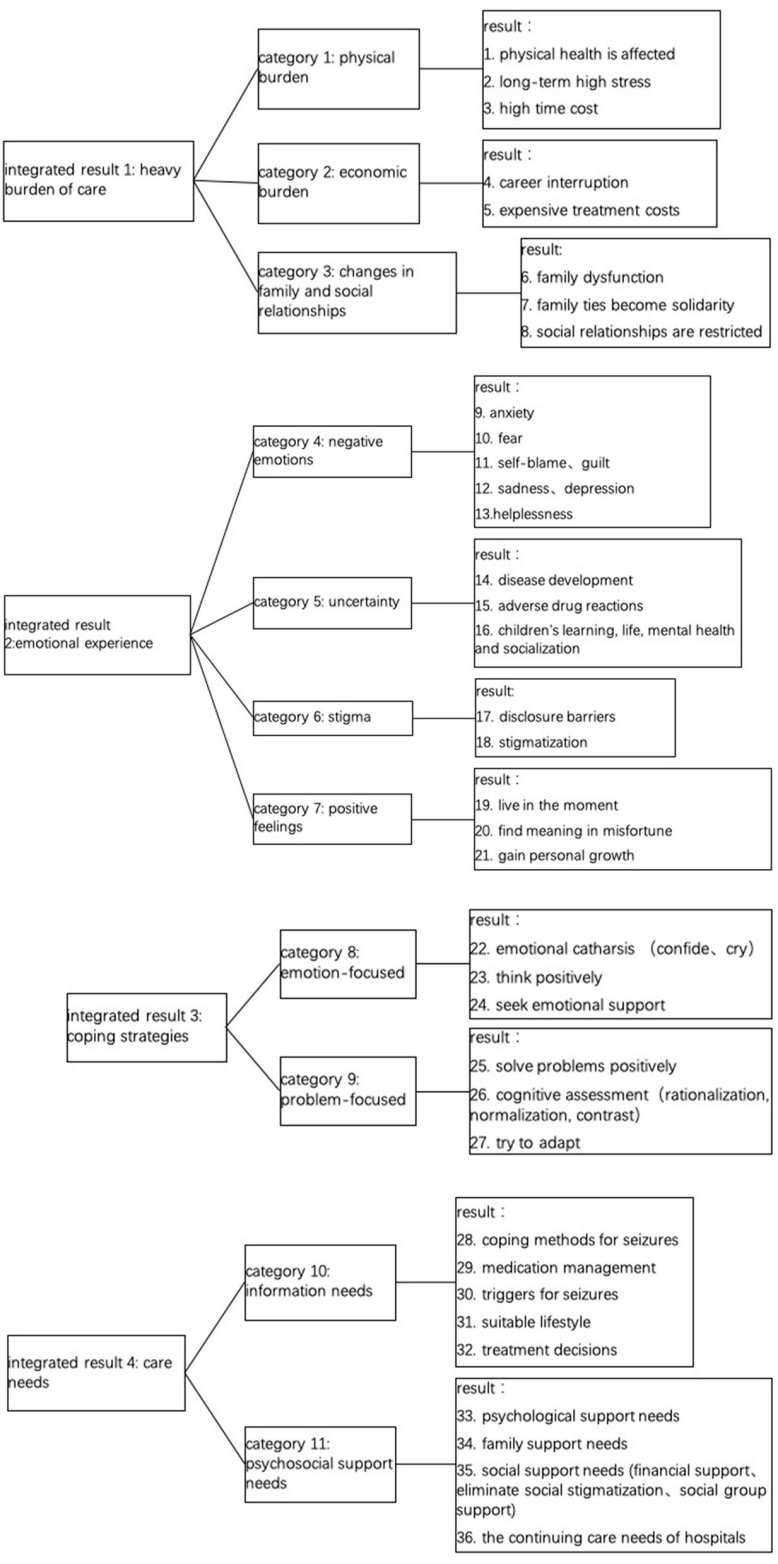
Research results, categories and integrated results of included studies.

The included studies covered a wide range of experiences of caregivers of children with epilepsy. 36 research results were extracted from these studies, which were combined into 11 categories, and finally formed 4 themes: (a) heavy burden of care; (b) emotional experience; (c) coping strategies; (d) care needs. Figure 2 shows the research results, categories and integrated results of included studies.

### Theme 1: Heavy burden of care

Almost all caregivers reported that the burden of caring for children with epilepsy was heavy. The burden included three aspects: physical health status, economic status and social life.

#### Category 1: Physical burden

As caregivers focused all their energy on the child, their own physical condition became overwhelmed. Long-term care for the child made the caregiver neglect their own health, and coupled with long-term mental stress, the caregiver's own health was threatened.

“*…We couldn't get a good rest…In order to take care of the children, my own illness dragged on for months?”* (12).

#### Category 2: Economic burden

Because of the specific nature of epilepsy, caregivers often needed to be around to monitor the condition of the child in order to cope with the sudden seizure, and many caregivers gave up their education and work, resulting in the interruption of their career development. In addition, epilepsy treatment cycle was long, and needed long-term medication, no source of income increased their economic burden.

“*When my wife realized my son was having seizures again, she left work and began spending more time to take care of him. If she didn't do that and was working instead we would have a better economic status.”* (22).

#### Category 3: Changes in family and social relationships

In the process of treating epilepsy, treatment decisions were involved. Differences in parental treatment decisions, coupled with physical stress, led to deteriorating family relationships and affected family functioning. Some caregivers were forced to give up socializing because of caring for the child. However some other caregivers showed family members had become more united in coping with epilepsy.

“*It's been a big impact on my husband and myself, our relationship…. We have arguments about everything from what to do with medications to what her schooling should be, to riding the bus to school, I mean…”* (13).

### Theme 2: Emotional experience

#### Catogory 4: Negative emotions

The caregivers' emotional experience was complex. Most caregivers had experienced negative emotions at one time. Some caregivers said they felt frightened and scared about the seizure because they didn't know how to deal with them. Some parents felt remorse and guilty because they believed they were the cause of their child's epilepsy. They worried that epilepsy could affect children's learning, social interaction and daily life, and they often felt anxious, sad and even hopeless.

“*The child's sudden delirium, convulsions, foaming at the mouth, scared me almost to faint.”* (12).

“*…I blamed myself. Why my child got this disease? Was it because I ate wrong food when I was pregnant? ”* (8).

“*I am worried about his future career. I'm worried that he cannot find a job due to his epilepsy. And he would not get equal treatment like others when he works in a company.”* (15).

#### Category 5: Uncertainty

The typical and unique experience of caregivers of children with epilepsy was the sense of uncertainty. They were full of uncertainty about the child's physical health, study, social life, daily life and future. They did not know whether the disease will affect the child's future study and work, which made them very anxious. The side effects of medication could add to the uncertainty.

“*See, my daughter is 10 and she started having seizures when she was 5 and, I guess the biggest impact on me would just be the stress of not knowing what was going on, what was going to happen to her, and how things were going to go, and why it happened, and then, just, worry for her, worry, about difficulties in school, and with friends and things like that.”* (13).

#### Category 6: stigma

In some countries and cultures, epilepsy was poorly understood and was often associated with social stigma. Some caregivers were even afraid to tell others about their child's seizure for fear of abuse, discrimination and isolation. Some caregivers were stigmatized as a result. They didn't know how to teach their children to deal with the stigma of epilepsy, and caregivers were struggling with stigma all the time. They longed for the understanding and support of society.

“*They looked us as if our child has AIDS, our child is sick and we are not responsible for it. I do not know why people look at me like that, it's so irritating that I want to die. It is so good that others are unaware of my child's disease.”* (16).

#### Category 7: Positive feelings

Some caregivers experienced positive feelings during the caring process. They chose to live in the present moment and tried to focus on the good aspects of the decease. They believed that their faith would help them get through it. They found value and positive meaning in their misfortune. In this process, they gained personal growth and the family relationship became more united.

“*We knew we were dealing with something that down the track will have a great outcome so really it was just a short-term issue that we're happy to deal with.”* (17).

### Theme 3: Coping strategies

#### Category 8: Emotion-focused coping strategies

Caregivers adopted different coping strategies to deal with problems in the caring process: problem-focused coping strategies and emotion-focused coping strategies. Caregivers who used emotion-focused coping would try to reduce the negative emotions associated with the problem, such as positive thinking and seeking emotional support. Most caregivers used emotion-focused coping strategies, such as crying to let their emotions out or talking about their pain with family and friends to reduce negative emotions. Or they tried to accept epilepsy, normalize it, live with it.

“*I talked to my neighbor when I was upset… Sometimes, she taught me how to handle my son when he has fits… Otherwise, I will call my mother. We talked about anything. Although I did not tell her much about my problem, I feel much better after talking to her*.” (15).

#### Category 9: Problem-focused coping strategies

Caregivers who used problem-focused coping would address the problem that caused the distress. They were active in solving problems and adapting to the status through reasonable cognitive assessment.

“*No, I'm not going to lay down and say it's all terrible and he's going to struggle with this; we'll do what we can to be pro-active about it.”* (17).

### Theme 4: Care needs

#### Category 10: Information needs

Caregivers expressed their unmet needs and desired for support from health workers and society. There was a common need for caregivers to acquire knowledge about the disease, such as how to deal with seizure, medication management, seizure triggers, and access to the right sources of information. Children with epilepsy were limited in diet and activity, and their caregivers had blurred boundaries. They urgently needed lifestyle guidance from professional.

“*…It would be a blessing if there were some kind of help from the school, the doctors, or anyone else to help me cope with the way I should handle epilepsy and my daughter's social life without being scared for her…”* (20).

“*The doctor said the child needed proper activities, but I don't know what he can do.”* (3).

There is a wide range of treatments methods to choose for caregivers, and caregivers are often indecisive in treatment decisions. They want professionals to help them make treatment decisions. They need continuity of care to ensure long-term outcomes. It may be helpful to establish care groups where caregivers can share information.

“*Because even if the doctor wants (to give more information about epilepsy), other patients are waiting outside… He is rushing… So, I am not satisfied. That's why, we should at least have a community… so that all parents can share their problems… I don't have anyone to share my problem with. My husband said it is fine to have a seizure. But what is the way to solve it? How to share?”* (15).

#### Category 11: Psychosocial support needs

There would be a lot of negative emotions in the process of care, and caregivers hoped to get professional psychological support. More importantly, caregivers wanted to gain social understanding and support, to be free from stigma, and to provide children with a good learning and living environment. In addition, financial support from the community, support from social groups and continuous care in hospitals were also necessary.

“*…I'm devastated. I feel like I can't hold on.”* (3).

“*I'm worried about who will support my child after me.”* (18).

## Discussion

This review addresses the experiences and needs of caregivers of children with epilepsy. Caregivers beared heavy burden of care, which made them generate a lot of negative emotions. The caregivers adopted different coping strategies, and a small number of caregivers could cope effectively, and found positive meaning of life in the process of caring. Disease-related knowledge were needed for caregivers to improve care capacity. Support from multiple levels, including the social level, school level and health care institution level were essential, to improve their existing difficulties.

In this review, a majority of caregivers reported heavy burden of care and negative emotional experiences, which was consistent with other studies that suggested that epileptic caregivers had a high burden of disease socially, emotionally, functionally, and economically (24). The long-term burden led to an increased prevalence of mental disorders among caregivers. Recent results showed that the prevalence of anxiety symptoms and depression in caregivers of children with epilepsy was 25.0 (25) and 23.5% (9), suggesting that healthcare providers should pay attention to the psychological and emotional symptoms of caregivers. Screening for mental health problems in caregivers should be incorporated in a family-centered approach to the management of childhood epilepsy. What's more, there is a need to identify the best ways of supporting caregivers of children with epilepsy who present with significant levels of mental health symptoms (26). However, there are currently few reported interventions that can reduce the burden on caregivers of children with epilepsy. Rabiei et al. (27) evaluated the effects of the Fordyce's 14 Fundamentals for Happiness Program on happiness and caregiver burden among caregivers of patients with epilepsy. Findings showed that this program significantly increased happiness and reduced caregiver burden. The Fordyce's 14 Fundamentals for Happiness Program could help individuals better understand their problems and more effectively cope with them through improving their logical thinking ability. Another study (28) evaluated the effectiveness of Web-based Epilepsy Education Program (WEEP), and the results showed that WEEP could improve caregivers' self-efficacy and reduce anxiety. WEEP could provide caregivers with information about epilepsy, treatment, and first aid, which was considered one of the most important components of epilepsy self-management to provide accurate, reliable, and accessible information sources that were not limited by time and space. Future research can explore the effectiveness and extensibility of these programs, and more effective interventions should be developed based on the actual situation.

Epilepsy has long been stigmatized, and people with epilepsy experience social stigma and discrimination in their daily lives. Stigma refers to a strong feeling of disapproval. Studies have shown that more than half of family members of patients with epilepsy experience stigma, and stigma negatively impacts caregivers' mental health, such as shame, low self-esteem, anger, and disorder disclosure (29). While interventions have been implemented to reduce epilepsy stigma at the public awareness level, policy-based level, school-based level, and targeted at people with epilepsy and their caregivers and peers level, stigma and discrimination remain widespread worldwide, and the lack of research on interventions to reduce stigma suggests an urgent need for more research, policies and actions (30).

Heavy burden of care affects caregivers' quality of life, but positive or adaptive coping strategies can improve caregivers' mental health status (31). Coping strategies can generally be divided into problem-focused coping and emotion-focused coping (32). In this review, most caregivers of children with epilepsy adopted emotion-focused coping strategies, such as catharsis and seeking emotional support, which was consistent with the research results of Hajisabbagh (33). Compared with emotion-focused coping, problem-centered coping was generally believed to improve caregivers' happiness (34, 35), but it could not be denied that emotion-focused coping could also help individuals adapt to stressful situations. Because emotional catharsis could prevent the accumulation of negative emotions, seeking emotional support could increase the resources to cope with stress. healthcare and social workers should help caregivers to develop effective coping strategies, and specific interventions are needed to be developed.

In this review, caregivers still had some unmet needs, including the need for disease information, psychological support and social support, etc. As far as we know, caregivers of children with epilepsy have insufficient information about epilepsy disorders. In one study, only 5% of caregivers knew the first-aid basics to apply in the case of a seizure (36). Unmet information needs led to greater stress, poorer psychosocial outcomes and lower satisfaction with healthcare services (37). There is a need for healthcare professionals to target information needs with interventions such as support groups and accessible websites. Establish a professional psychological support team to carry out continuous care for caregivers, and intervene as early as possible for caregivers with mental disorders. In addition, social support is also necessary. The government should take measures to reduce the stigma of epilepsy and provide a convenient medical environment for families of epilepsy to reduce the burden on caregivers.

### Limitations

This review has some limitations. First of all, we only searched eight databases and did not search gray literature, and due to the limitation of language, we only searched and included relevant literatures in both Chinese and English, so some literatures might be omitted from this review. Secondly, the included studies may not represent all the caregivers of children with epilepsy, because some children with epilepsy have more severe symptoms or multiple comorbidities, leading to differences in the experience of caregivers. Therefore, to some extent, the comprehensiveness and objectivity of integration may be affected. Despite its limitations, this review integrates the available literature on the experiences of caregivers of children with epilepsy and has implications for further understanding of the caregivers' experiences.

### Implications on future research and practice

Although the caregiver burden of epilepsy is recognized, existing reports of effective interventions are insufficient. Future research could develop interventions to reduce the caregiver burden and improve their mental health. In addition, health care institutions need to take steps to make disease information more accessible to caregivers and enhance caregivers' ability to care. The society needs to increase investment and make policies to improve the medical environment for families of epilepsy and eliminate the social stigma of epilepsy.

## Conclusion

This review synthesized the available evidence on the experiences and needs of caregivers of children with epilepsy. The caregiver burden and psychological stress were enormous and their care needs were unmet. Healthcare workers need to develop interventions to reduce the burden of care, improve mental health status, provide disease-related information and enhance their caring capacity. Society needs to take measures for children with epilepsy and their families to create a friendly medical environment and free from stigma living environment.

## Data availability statement

The original contributions presented in the study are included in the article/supplementary material, further inquiries can be directed to the corresponding author.

## Author contributions

ZY made substantial contributions to conception and design, acquisition of data, analysis, and interpretation of data. ZY, QS, KH, and YW were involved in drafting the manuscript and making critical changes to important intellectual content. ZY and QS participated sufficiently in the work to take public responsibility for appropriate portions of the content. QS, KH, YW, and XS agreed to be accountable for all aspects of the work in ensuring that questions related to the accuracy or integrity of any part of the work are appropriately investigated and resolved. All authors contributed to the article and approved the submitted version.

## Conflict of interest

The authors declare that the research was conducted in the absence of any commercial or financial relationships that could be construed as a potential conflict of interest.

## Publisher's note

All claims expressed in this article are solely those of the authors and do not necessarily represent those of their affiliated organizations, or those of the publisher, the editors and the reviewers. Any product that may be evaluated in this article, or claim that may be made by its manufacturer, is not guaranteed or endorsed by the publisher.
